# Unique allergen-specific human IgE monoclonal antibodies derived from patients with allergic disease

**DOI:** 10.3389/falgy.2023.1270326

**Published:** 2023-10-12

**Authors:** Bryan R. E. Smith, Kristina Reid Black, Max Bermingham, Sayeh Agah, Jill Glesner, Serge A. Versteeg, Ronald van Ree, Glorismer Pena-Amelunxen, Lorenz Aglas, Scott A. Smith, Anna Pomés, Martin D. Chapman

**Affiliations:** ^1^ InBio, Charlottesville, VA, United States; ^2^ InBio, Cardiff, United Kingdom; ^3^ Department of Experimental Immunology, Amsterdam University Medical Center, Amsterdam, Netherlands; ^4^ Department of Biosciences and Medical Biology, University of Salzburg, Salzburg, Austria; ^5^ Department of Pathology, Microbiology and Immunology, Vanderbilt University Medical Center, Nashville, TN, United States

**Keywords:** IgE antibodies, allergy diagnostics, food allergy, alpha-gal, allergenic epitopes

## Abstract

**Introduction:**

Allergic reactions are mediated by human IgE antibodies that bind to and cross-link allergen molecules. The sites on allergens that are recognized by IgE antibodies have been difficult to investigate because of the paucity of IgE antibodies in a human serum. Here, we report the production of unique human IgE monoclonal antibodies to major inhaled allergens and food allergens that can be produced at scale in perpetuity.

**Materials and methods:**

The IgE antibodies were derived from peripheral blood mononuclear cells of symptomatic allergic patients, mostly children aged 3–18 years, using hybridoma fusion technology. Total IgE and allergen-specific IgE was measured by ImmunoCAP. Their specificity was confirmed through ELISA and immunoblotting. Allergenic potency measurements were determined by ImmunoCAP inhibition. Biological activity was determined *in vitro* by comparing *β*-hexosaminidase release from a humanized rat basophilic cell line.

**Results:**

Human IgE monoclonal antibodies (*n* = 33) were derived from 17 allergic patients with symptoms of allergic rhinitis, asthma, atopic dermatitis, food allergy, eosinophilic esophagitis, or red meat allergy. The antibodies were specific for five inhaled allergens, nine food allergens, and alpha-gal and had high levels of IgE (53,450–1,702,500 kU/L) with ratios of specific IgE to total IgE ranging from <0.01 to 1.39. Sigmoidal allergen binding curves were obtained through ELISA, with low limits of detection (<1 kU/L). Allergen specificity was confirmed through immunoblotting. Pairs of IgE monoclonal antibodies to Ara h 6 were identified that cross-linked after allergen stimulation and induced release of significant levels of *β*-hexosaminidase (35%–80%) from a humanized rat basophilic cell line.

**Conclusions:**

Human IgE monoclonal antibodies are unique antibody molecules with potential applications in allergy diagnosis, allergen standardization, and identification of allergenic epitopes for the development of allergy therapeutics. The IgE antibody probes will enable the unequivocal localization and validation of allergenic epitopes.

## Introduction

The discovery of IgE transformed the investigation of allergic diseases and led to the development of *in vitro* diagnostic tests which have become a routine part of patient care (1–4). Measurements of total IgE and allergen-specific IgE antibodies as markers of allergic responses have had a tremendous impact. They have fueled clinical trials of subcutaneous and oral immunotherapy and led to the introduction of new treatment modalities, such as monoclonal anti-IgE therapy and biologics for treatments of asthma and atopic dermatitis, and early introduction of foods for prevention of food allergy (5, 6). Analysis of IgE antibody responses has played a key role in investigating the mechanisms of anaphylaxis, the structural biology of allergens, and standardization of allergenic products (7). IgE sensitization has been a key indicator for epidemiologic and population-based studies of the etiology of allergic diseases (8–11).

One question that has eluded allergists since the discovery of IgE has been “What do IgE antibodies recognize and what constitutes an ‘allergenic’ epitope?” (12). Indeed, this question has been somewhat of a holy grail among the allergy community and has been difficult to resolve. Natural IgE antibody responses to environmental allergens are polyclonal, and the concentrations of the IgE antibody in the serum are very low, typically <100 or <250 ng/ml. These limitations exclude the use of natural IgE antibodies for direct structural studies of allergen–antibody interactions using x-ray crystallography and/or nuclear magnetic resonance (NMR) spectroscopy (13).

Alternatively, researchers have used indirect approaches to identify conformational IgE epitopes, namely, inhibition of IgE antibody binding to structurally modified allergens, using anti-peptide antibodies to inhibit IgE binding, or using hydrogen/deuterium exchange in NMR to generate variants with reduced IgE binding (13, 14). Linear IgE epitopes on food allergens have been defined using direct peptide-binding assays (15–17). Conformational epitopes may also be important for some food allergens (18).

The recent development of the hybridoma technology to produce allergen-specific human IgE monoclonal antibodies (hIgE mAb) provides a strategic pathway to investigate IgE responses (12, 19). The technology was first used to produce hIgE mAb to *Aspergillus fumigatus* from patients with allergic bronchopulmonary aspergillosis (19). An advantage of using the hybridoma technology is that milligram (mg) quantities of allergen-specific hIgE mAb can be produced with a natural pairing of heavy and light chains (12). Here, we describe the derivation of unique panels of hIgE mAb from children and adults with allergic rhinitis, asthma, atopic dermatitis, eosinophilic esophagitis, and adverse reactions to foods or red meat allergy. The hIgE mAb recognized the major inhaled allergens and food allergens of diagnostic and therapeutic importance, such as alpha-gal, the carbohydrate associated with red meat allergy (20).

## Materials and methods

### Generation of allergen-specific hIgE mAb

Peripheral blood mononuclear cells (PBMC) were obtained from 17 allergic subjects, aged 3–63 years, recruited from within the Vanderbilt University Medical Center (VUMC). The subjects diagnoses were based on clinical history, *in vitro* IgE testing, and/or skin prick tests to inhaled allergens or food allergens (Table 1). The protocol for recruiting and collecting blood samples from allergic subjects was approved by the VUMC Institutional Review Board (IRB 141330 and 142030).

**Table 1:** Subject demographics and clinical information.

Subject	sIgE mAb (specificity)	Age	Sex	Allergic diseases	IgE (kU/L)	Allergen hIgE (kU/L)	SPT (mm wheal)	IgE B-cell frequency
P1	4G4 (Ara h 1)	18	M	AF, Asthma, AD	677	Peanut: 65	Peanut: 20 × 25 HDM *D. f*.: 15 × 10 HDM *D. pt*.: 10 × 8	12
	38B7 (Ara h 2)							
	13D9 (Ara h 2)							
	16A8 (Ara h 2)							
	3C3 (Ara h 3)							
	8F3 (Ara h 6)							
	7B6 (Ara h 6)							
	15C2 (Ara h 6)							
	21C10 (Ara h 6)	** **						
	1B8 (Der p 2)							
	2F10 (Der p 2)							
	13B6 (Can f 6)							
P2	1D5 (Bos d 8)	3	F	AF, AD, AR	ND	Cow's milk: >100	Dog: 8 × 16	4
	11B6 (Gal d 2)					Whole egg: >100	Cat: 5 × 14	
	2F5 (Ana o 3)					Cashew: >100	HDM *D. f.*: 9 × 16	
P3	2L11 (Der p 1)	59	F	Asthma, AF, AR	1,396	HDM *D. pt.*: 6.7	ND	11
	1J11 (Can f 1)					Dog: >100		
						Cat: >100		
P4	16D9 (alpha-gal)	63	F	AF, AR	1,063	Alpha-gal: 86	ND	2
	10H8 (alpha-gal)							
P5	6A1 (Fel d 1)	10	M	Asthma, AF, EoE,	ND	ND	Cat: 15 × 30	9
	1B7 (Fel d 1)			AR				
P6	1C14 (Der p 1)	62	M	Asthma, AR	>5,000	HDM *D. pt.*: >100	ND	
P7	26C3 (Ara h 2)	15	M	AF, Asthma	1,615	Peanut: >100	ND	6
P8	20G11 (Ara h 6)	6	F	AF	351	Peanut: >100	Peanut: 24 × 45	2
P9	14G12 (Ara h 3)	10	M	AF, Asthma	ND	Peanut: >100	Peanut: 24 × 24	8
P10	9H7 (Jug r 1)	6	F	AF, EoE	ND	ND	Walnut: 8 × 6	11
P11	1E7 (Gal d 4)	5	M	AF, AD	ND	Peanut: >100	Peanut: 16 × 10	7
P12	2G1 (Der p 2)	8	M	AF, Asthma, AR	ND	ND	HDM *D. pt.*: 10 × 10	10
P13	11A12 (Fel d 1)	5	M	AF, Asthma	ND	ND	Cat: 6 × 15	8
P14	3B10 (Ara h 1)	9	F	AF	ND	Peanut: >100	Peanut: 19 × 40	2
P15	9H11 (Ara h 2)	27	M	AF	ND	ND	ND	3
P16	11F10 (Ara h 2)	10	M	AF, Asthma, AD	802	Peanut: 73	Peanut: 8 × 11	7
P17	20M10 (Ara h 6)	13	F	AF, Asthma, AR	396	229	Peanut: 45 × 30	9

AF, adverse food reaction; AD, atopic dermatitis; AR, allergic rhinitis; EoE, eosinophilic esophagitis; SPT, skin prick test; ND, not determined; HDM, house dust mite; sIgE, specific IgE.

Subject age, sex, allergy history, IgE, and allergen-specific IgE are shown. IgE B-cell frequency is the number of IgE-positive cells per 10 million PBMC.

Detailed cell culture methods for producing hIgE mAb from PBMC have recently been published (12, 19, 21, 22). Full technical details of these methods can be found at the following link: https://insight.jci.org/articles/view/123387/sd/1 (accessed 110923) and can be downloaded as a PDF file. Briefly, PBMC were cultured for 7 days with irradiated NIH3T3 cells expressing CD154, B-cell-activating factor, and IL-21 to selectively expand B cells. After screening for IgE through ELISA, IgE-producing B cells were fused with non-secreting myeloma cells and cloned using single-cell flow cytometry. The hIgE mAb generated using this B-cell culture system and hybridoma methodology were not created by artificial class switching. Screening for specificity only occurred after all IgE lines were fused. The hIgE mAb were screened for allergen specificity through ELISA by using microtiter plates coated with ∼1 µg/well of purified natural or recombinant allergens: nDer p 1, nDer p 2, nFel d 1, nCan f 1, rCan f 6, nAra h 1, nAra h 2, nAra h 3, nAra h 6, nBos d 5, nBos d 11, nGal d 2, nGal d 4, rAna o 3, and rJug r 1. The bound hIgE mAb was detected with peroxidase-labeled mouse anti-human IgE. The hIgE mAb were purified by affinity chromatography using immobilized monoclonal anti-IgE antibody omalizumab. The purity of the hIgE mAb was assessed by SDS-PAGE under reducing conditions.

### Quantification and specificity of the hIgE mAb

The concentration of total IgE and allergen-specific IgE was determined through ImmunoCAP (Thermo Fisher Scientific, Kalamazoo, MI, USA, capacity) (4, 23). The specificity of the hIgE mAb was assessed through ELISA by using purified natural or recombinant allergens and immunoblotting. ImmunoCAP inhibition experiments were used to assess the ability of the hIgE mAb to inhibit polyclonal IgE antibody binding to allergen extracts.

The IgE concentration of the purified hIgE mAb was initially determined by total IgE ImmunoCAP using highly diluted samples (1/1,000 to >1/20,000). Once the concentration of each purified hIgE mAb stock was determined, samples were diluted to a concentration of ∼50,000 kU/L and re-assayed at 1/100–1/10,000 dilutions. The samples were assayed for the concentration of the allergen-specific IgE antibody by ImmunoCAP, and the ratio of total IgE to allergen-specific IgE was compared.

The specificity of the hIgE mAb was further assessed by ELISA using microtiter plates coated with 100 ng/well of purified allergen. Doubling dilutions of the hIgE mAb were prepared over a three-log range (0.1–100 kU/L) to form dose–response curves. The bound hIgE mAb was detected using peroxidase-labeled goat anti-human IgE (SeraCare, Milford, MA, USA).

For immunoblotting, purified natural or recombinant allergens, cetuximab, and extract samples were run on SDS-PAGE under reducing or non-reducing conditions. Allergens were transferred to PVDF membranes and incubated with a hIgE mAb at 1–4 µg/ml for 1 h. The membranes were washed and incubated for 30 min with alkaline phosphatase-conjugated mouse anti-human IgE Fc (SouthernBiotech, Birmingham, AL, USA) and Strep-Tactin (Bio-Rad, Hercules, CA, USA). They were developed with chemiluminescent substrate prior to chemiluminescence imaging.

### ImmunoCAP inhibition

ImmunoCAP inhibition was performed according to the manufacturer's instructions. Pools of the hIgE mAb for Ara h 1 and Ara h 2 and for Der p 1 and Der p 2 were compared with human serum pools from patients allergic to peanuts and to house dust mites, respectively. Pools were diluted to ∼10 kU/L for peanut and house dust mite ImmunoCAP, respectively. The pool of hIgE mAb for peanuts comprised six Ara h 2 hIgE mAb (9H11, 13D9, 11F10, 38B7, 26C3, and 16A8) and a single Ara h 1 hIgE mAb (4G4). The pool of hIgE mAb for house dust mites used a single Der p 1 hIgE mAb (2L11) and three Der p 2 hIgE mAb (2G1, 1B8, and 2F10). Sera from patients allergic to peanuts were provided by Dr. Stephen Dreskin of the University of Colorado, Denver, CO, USA (24). Sera from patients allergic to dust mites were obtained from the CREATE project (25). Human serum samples were diluted to ∼10 kU/L against the allergen extract for use in ImmunoCAP inhibition. Allergen extracts from house dust mites or peanuts were diluted in serial 10-fold dilutions from 1,000 to 0.01 µg/ml and were used to inhibit pools of hIgE mAb or pools of allergic serum to the allergen ImmunoCAP.

### Biological activity of the hIgE mAb

The functional activity of the hIgE mAb was assessed by using humanized rat basophilic leukemia (huRBL) cells transfected with human FcɛRI for passive sensitization with hIgE mAb (RBL-2H3 cell line) (26, 27). *β*-Hexosaminidase release was used as a marker of allergen-induced degranulation. Briefly, RBL-2H3 cells were sensitized with hIgE mAb pairs to Ara h 6 at 5–30 kU/L. After overnight sensitization with hIgE mAb, the cells were stimulated with 50 ng allergen. Untreated cells and cells sensitized with hIgE mAb but not stimulated with allergen were used as controls. For data analysis, the average background signal was subtracted from each value, the percentage of maximal lysis was calculated, and min–max normalized values were plotted. Results were observed from three independent experiments. An ordinary one-way ANOVA with a Tukey's mean multiple comparisons analysis was used to determine significant differences between the mediator release produced by all the combinations.

## Results

### Subject demographics and clinical information

The hIgE mAb to inhaled and food allergens were derived from 33 fusions from 17 selected donors who presented to the VUMC allergy clinic with clinical histories of allergic rhinitis, asthma, atopic dermatitis, eosinophilic esophagitis, or adverse reactions to foods. The donor population included 13 children (aged 3–18 years) and four adults (aged 27–63 years), with a preponderance of male (*n* = 10) to female (*n* = 7) donors. The research subjects had total IgE levels of 351 to >5,000 kU/L, together with high levels of allergen-specific IgE (65 to >100 kU/L) and/or strongly positive skin prick tests to allergen extracts (Table 1). More than 50% of the hIgE mAb were derived from three subjects with multiple sensitivities (P1–P3, Table 1), including a 3-year-old child allergic to food from whom the hIgE mAb to cashew, milk, and egg allergens were derived. The IgE B-cell frequency, as measured by the number of IgE-positive B-cell culture wells per 10 million PBMC used in culture, ranged from 2 to 12 per 10^7^ PBMC, which is comparable to that reported in recent studies of IgE primary B-cell cultures from *Aspergillus* or parasitic worm-infected donors (19, 22).

### Quantitative comparison of hIgE mAb reactivity

**Figure 1:**
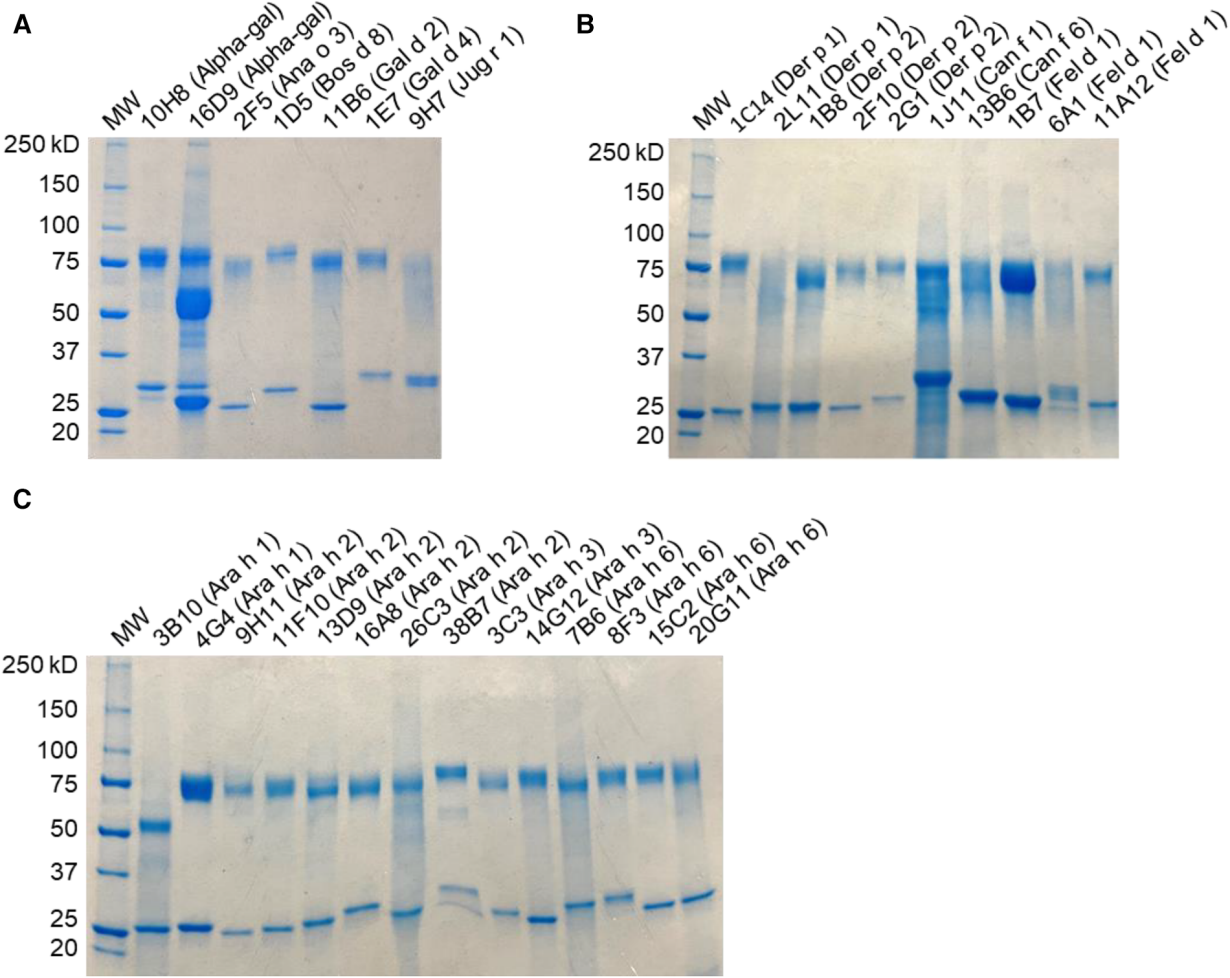
SDS-PAGE analysis of hIgE mAb. Samples were run under reducing conditions and stained with Coomassie blue, indicating heavy- and light-chain bands. (**A**) Food allergen panel. (**B**) Inhaled allergen panel. (**C**) Peanut allergen panel.

This paper describes the IgE-binding activity and biological activity of 33 hIgE mAb directed against five inhaled allergens (Der p 1, Der p 2, Fel d 1, Can f 1, and Can f 6) and against nine food allergens (Ara h 1, Ara h 2, Ara h 3, Ara h 6, Bos d 8, Gal d 2, Gal d 4, Ana o 3, and Jug r 1) and alpha-gal. Multiple hIgE mAb were generated against several allergens such as Der p 1, Der p 2, Fel d 1, Ara h 1, Ara h 2, Ara h 3, and Ara h 6 and alpha-gal. Following monoclonal anti-IgE affinity purification, yields of hIgE mAb were typically 2–30 mg, and purity was >90% under reducing conditions on SDS-PAGE (Figure 1). Most of the hIgE mAb (*n* = 29) showed homogeneous heavy- and light-chain bands of 75 kD and 25–30 kD, respectively. Two hIgE mAb, to alpha-gal (16D9) or Ara h 1 (3B10), showed truncated heavy chains at ∼50 kD either alone or in addition to the 75 kD heavy chain. The IgE mAb 16D9 bound to alpha-gal and to FcɛRI by ELISA. Despite the presence of only the truncated form in 3B10, this antibody did specifically recognize Ara h 1. However, it did not bind to FcɛRI (data not shown), which indicated that this band with lower MW than expected for a heavy chain possibly results from a truncated form that lost its capacity to bind the IgE high-affinity receptor, while keeping its capacity to bind the allergen.

The total IgE measurements of purified hIgE mAb ranged from 53,450 to 1,702,500 kU/L (Table 2). To standardize IgE concentrations, the hIgE mAb were adjusted to a concentration of ∼50,000 kU/L and assayed for specific IgE using allergen extract or purified allergen ImmunoCAP. The ratio of total IgE to specific IgE varied by allergen and by individual hIgE mAb and ranged from <0.01 to 1.39 (Table 2). Low ratios (<0.2) were obtained for hIgE mAb to alpha-gal and to Ara h 6 (20M10), Can f 6, and Der p 1. Overall, a good agreement between the allergen-specific IgE values obtained with allergen extracts and purified allergens was reported.

**Table 2:** Total and allergen-specific IgE concentrations of hIgE mAb.

Allergen	hIgE mAb	Total IgE stock (kU/L)	Total IgE aliquot (kU/L)	Specific IgE aliquot (kU/L)	Extract IgE aliquot (kU/L)	IgE ratio specific: total	IgE ratio extract: total
Alpha-gal	10H8	78,125	51,800	6,500	^a^	0.13	^a^
	16D9	134,925	53,400	10,360	^a^	0.19	^a^
Ana o 3	2F6	1,139,500	57,400	37,067	46,443	0.65	0.81
Ara h 1	3B10	191,625	48,300	55,300	51,100	1.14	1.06
	4G4	614,750	53,967	49,400	39,300	0.91	0.73
Ara h 2	9H11	349,350	49,750	24,300	28,000	0.49	0.56
	13D9	309,725	50,550	69,000	40,900	1.36	0.81
	11F10	439,825	49,200	49,250	51,950	1.00	1.06
	38B7	107,750	48,350	31,950	49,800	0.66	1.03
	26C3	251,950	51,350	37,200	32,000	0.72	0.62
	16A8	1,702,500	58,417	28,233	32,150	0.48	0.55
Ara h 3	3C3	358,765	40,000	29,845	34,100	0.75	0.85
	14G12	230,300	48,750	40,900	155	0.84	0.00
Ara h 6	7B6	286,050	41,150	21,130	23,860	0.51	0.58
	8F3	500,000	40,300	36,250	33,365	0.91	0.83
	15C2	149,100	49,683	40,933	39,900	0.82	0.80
	20G11	334,875	54,000	47,985	42,235	0.89	0.78
	20M10	392,675	45,450	1,530	42,350	0.03	0.93
	21C10	406,175	54,200	49,000	40,550	0.90	0.75
Bos d 8	1D5	53,450	44,733	41,600	38,775	0.93	0.87
Gal d 2	11B6	129,900	66,950	81,083	90,667	1.21	1.35
Gal d 4	1E7	1,447,000	50,567	15,700	17,817	0.31	0.35
Jug r 1	9H7	340,500	47,367	30,003	25,513	0.63	0.54
Can f 1	1J11	341,000	59,000	20,600	14,450	0.35	0.24
Can f 6	13B6	119,767	52,567	5,333	3,223	0.10	0.06
Der p 1	1C14	315,567	54,867	328	169	0.01	0.00
	2L11	300,667	59,000	4,590	990	0.08	0.02
Der p 2	2G1	116,067	71,967	37,575	44,225	0.52	0.61
	2F10	221,050	46,483	19,925	19,925	0.43	0.43
	1B8	192,167	40,500	56.300	2,107	1.39	0.05
Fel d 1	6A1	353,550	63,800	14,933	25,600	0.23	0.40
	1B7	1,663,000	67,117	18,033	12,967	0.27	0.19
	11A12	525,000	59,967	52,667	39,383	0.92	0.69

^a^ Extract CAPs not available for alpha-gal.

Some hIgE mAb to peanut reacted strongly with allergen extract but displayed low IgE binding to the purified allergen, suggesting that the recombinant allergen(s) coupled to the cellulose solid phase of the ImmunoCAP may lack certain IgE epitope(s). This was investigated by comparing hIgE mAb binding to purified natural and recombinant allergens using the streptavidin CAP (28, 29). Purified nAra h 2, nAra h 3, and nAra h 6 were coupled to streptavidin CAPs and used to compare binding of four hIgE mAb. The hIgE mAb to Ara h 3 (14G12) and Ara h 6 (20M11) bound strongly to peanut extract but weakly (<10% reactivity) to the recombinant allergen ImmunoCAP. In contrast, the binding of these hIgE mAb to natural allergen on streptavidin CAP was 18–42-fold higher than the binding of hIgE mAb to the recombinant allergens and even exceeded its binding to peanut ImmunoCAP (Table 3).

**Table 3:** Comparison of IgE reactivity of hIgE mAb with natural and recombinant peanut allergens.

Allergen	hIgE mAb	ImmunoCAP	Conc. (kU/L)
Ara h 3	14G12	Peanut (f13)	31,150
		Ara h 3 (f424)	1,900
		nAra h 3^a^	35,150
Ara h 6	20M10	Peanut (f13)	42,350
		Ara h 6 (f447)	1,530
		nAra h 6^a^	63,500
Ara h 2	38B7	Peanut (f13)	67,600
		Ara h 2 (f423)	46,950
		nAra h 2^a^	79,000
		rAra h 2^a^	38,337
Ara h 2	13D9	Peanut (f13)	24,657
		Ara h 2 (f423)	62,350
		nAra h 2^a^	57,150
		rAra h 2^a^	8,115

^a^ Streptavidin CAP coupled with biotinylated natural Ara h 2, Ara h 3, or Ara h 6 or to recombinant Ara h 2.

### Specificity of the hIgE mAb

**Figure 2:**
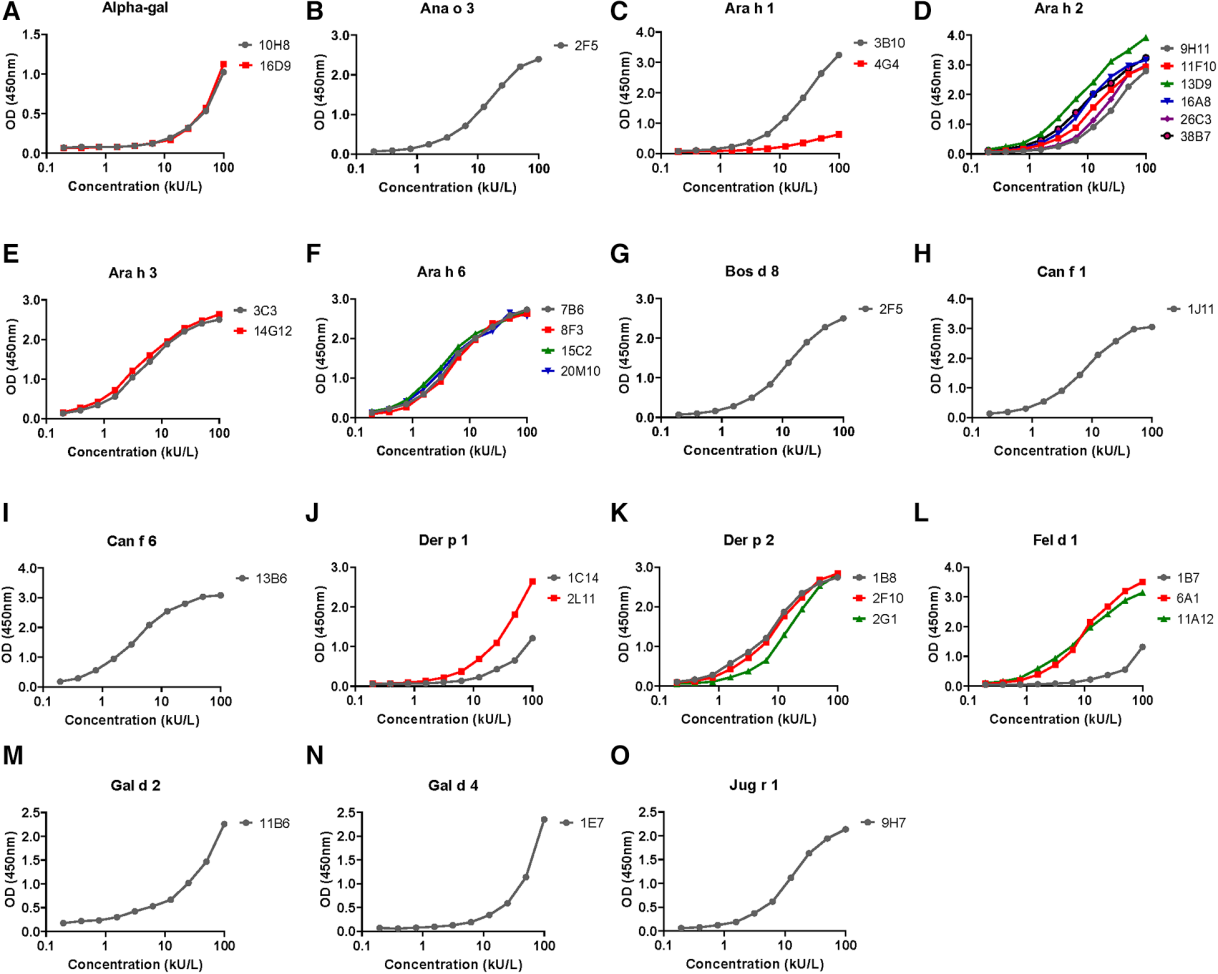
ELISA dose–response curves for hIgE mAb titered against purified natural or recombinant target allergen adsorbed onto microtiter plates (100 ng per well). Serial twofold dilutions of hIgE mAb were compared, starting at a concentration of 100 kU/L. The dose response curves for each of the 15 allergens tested are labeled (**A**) through (**O**), with insets denoting the allergen specificity e.g. A Alpha-gal, B Ana o 3, etc. The side bars indicate the individual hIgE mAb used for the control curves and are color coded to distinguish multiple antibodies. The ELISA curves are representative of two to three experiments performed for each allergen.

**Figure 3:**
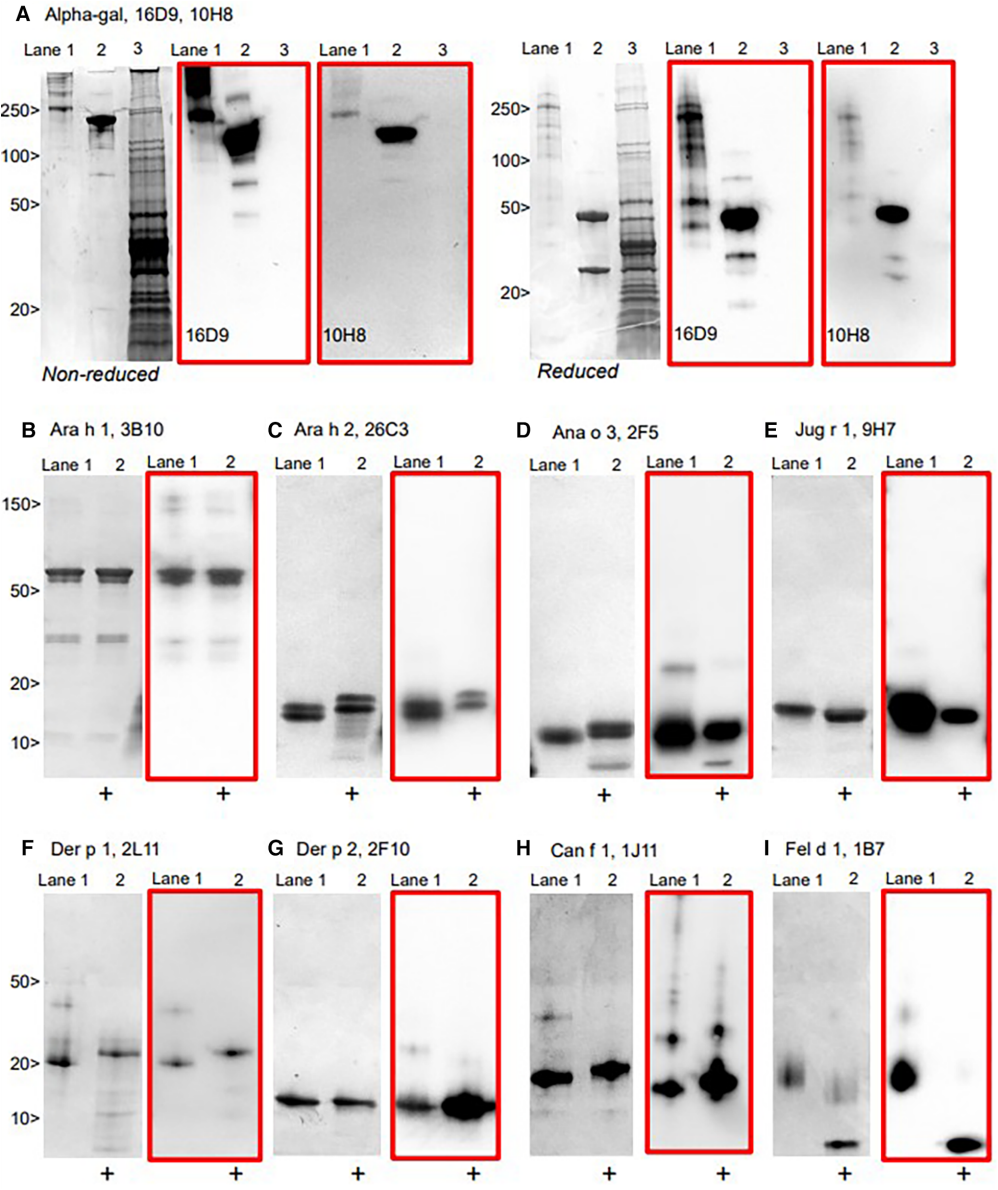
Immunoblotting of representative hIgE mAb using allergens run under non-reducing and reducing conditions on SDS-PAGE, with the matched immunoblot in red. (**A**) hIgE mAb to alpha-gal demonstrating reactivity to bovine thyroglobulin (Lane 1) and cetuximab (Lane 2), but not to chicken meat extract (Lane 3). (**B**–**I**) purified allergens non-reduced (Lane 1) and reduced [+] (Lane 2), with immunoblot (in red).

The hIgE mAb showed a strong binding to purified allergens in ELISA with dose–response curves from <1 to 100 kU/L (Figure 2). In most cases, the dose–response curves were sigmoidal with limits of detection of <1 kU/L. The specificity of the selected hIgE mAb to alpha-gal, food allergens, and inhaled allergens was further evaluated by immunoblotting using purified allergens and allergen extracts under reducing and non-reducing conditions (Figure 3). The two hIgE mAb to alpha-gal (16D9 and 10H8) showed strong reactivity and similar dose–response curves in ELISA using a purified bovine thyroglobulin. They also showed a strong binding to bovine thyroglobulin and to cetuximab by immunoblotting, and no reactivity to a chicken meat extract control (Figure 3A). Anti-Ara h 1 hIgE mAb 3B10 reacted strongly with the 60 kD sub-unit of Ara h 1, and the hIgE mAb to Ara h 2 (26C3) bound to both 17 kD bands in the Ara h 2 doublet (Figure 3B,C). The hIgE mAb to Ana o 3 (2F5) and to Jug r 1 (9H7) bound strongly to each respective allergen under non-reducing and reducing conditions (Figure 3D,E). For inhaled allergens, the hIgE mAb to Der p 1 (2L11), Der p 2 (2F10), and Can f 1 (1J11) bound predominantly to single bands at their respective molecular weights (Figure 2F–H). The hIgE mAb to Fel d 1 (1B7) bound to the 14 kD Fel d 1 homodimer under non-reducing conditions and to the ∼6 kD homodimer fragment under reducing conditions (Figure 3I).

### ImmunoCAP inhibition

**Figure 4:**
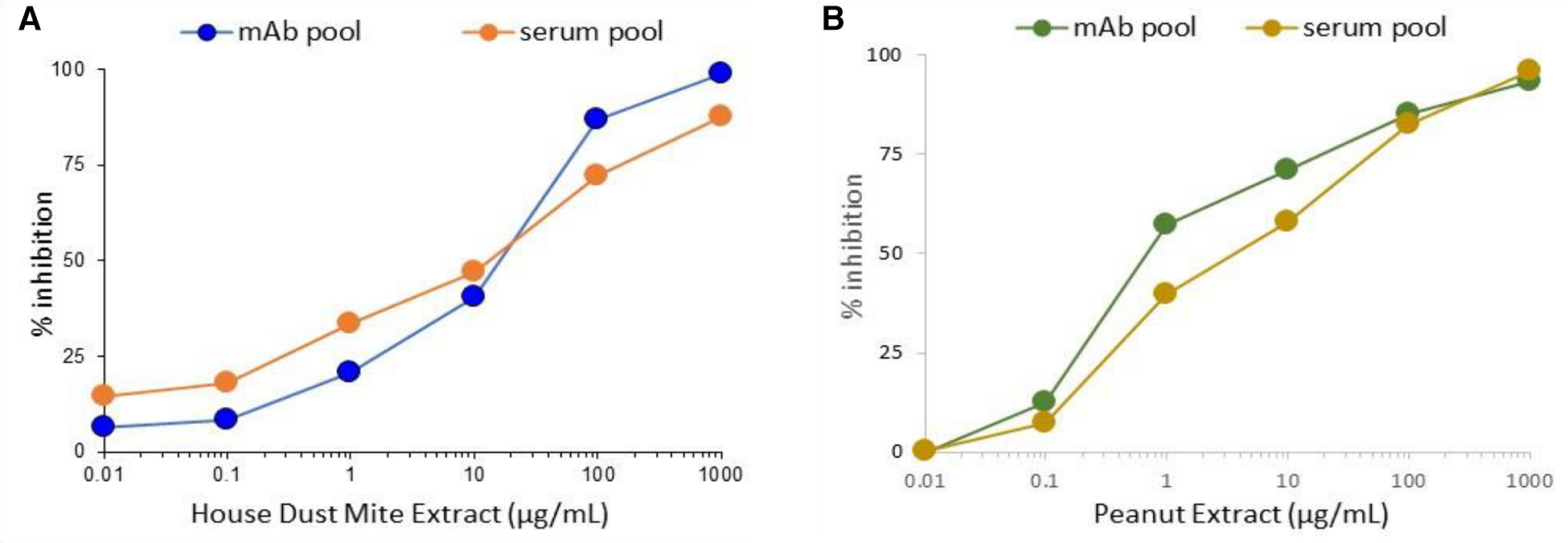
Comparison of inhibition of hIgE mAb or human serum pool IgE to house dust mite (**A**) or peanut extract (**B**).

To evaluate whether the pools of hIgE mAb could replace variable patient serum pools for potency assessment of immunotherapy extracts in standardization protocols, both were compared by ImmunoCAP inhibition targeting house dust mites and peanuts. Extracts of both allergen sources gave very similar inhibition curves using the pools of monoclonal antibodies and the pools of the human serum (Figure 4).

### Basophil mediator release by the hIgE mAb

The hIgE mAb to Der p 2, Fel d 1, or Ara h 2 were screened in pairs for their biological activity using a basophil mediator release assay by passively sensitizing huRBL cells. The aim was to determine (i) whether the hIgE mAb were directed against different epitopes and (ii) whether they could cross-link the high-affinity human IgE Fc*ε* receptor that was transfected into the RBL cell line upon allergen stimulation to induce degranulation. The mediator release data for hIgE mAb pairs to Der p 2, Fel d 1, and Ara h 2 was recently published (27). For each of the selected allergens, antibody pairs induced 40%–80% *β*-hexosaminidase release, providing a strong evidence for multiple non-overlapping IgE epitopes.

**Figure 5:**
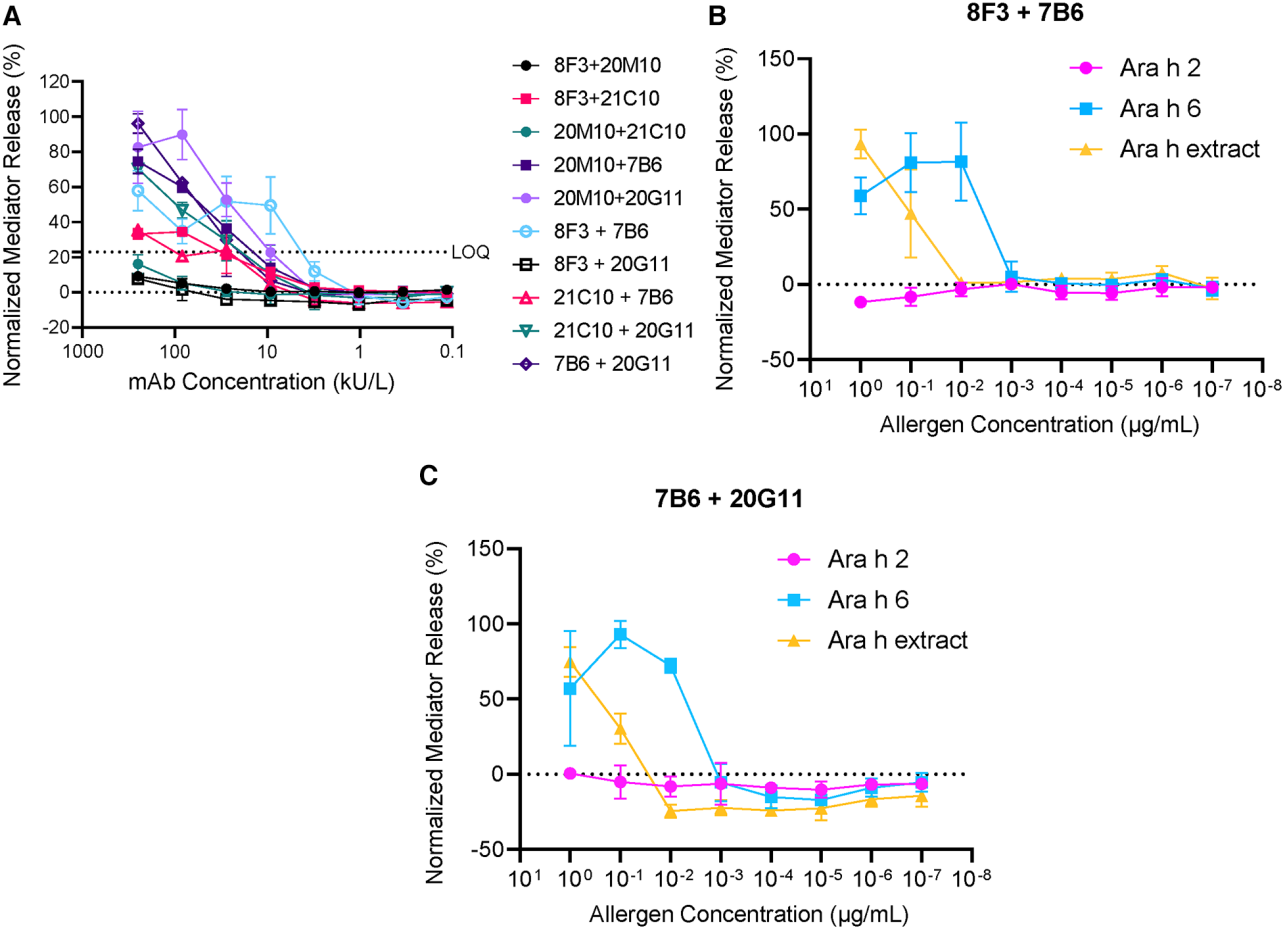
Mediator release induced by hIgE mAb to Ara h 6. (**A**) Dose–response curves using 10 different combinations of hIgE mAb. (**B**) Comparison of mediator release using Ara h 6, Ara h 2, and Ara h extract (8F3 and 7B6). (**C**) Comparison of mediator release using Ara h 6, Ara h 2, and Ara h extract (7B6 and 20G11). LOQ, limit of quantification.

In the present studies, five hIgE mAb to Ara h 6 (8F3, 20M10, 21C10, 20G11, and 7B6) were analyzed for basophil mediator release. Dose–response curves showed 30%–90% *β*-hexosaminidase release using several combinations of the hIgE mAb (Figure 5). Two of the hIgE mAb pairs (8F3 + 7B6 and 7B6 + 20G11) showed 50%–90% *β*-hexosaminidase release with Ara h 6 and with peanut extract, but no release with Ara h 2 was determined, indicating that they were specific for Ara h 6. The data strongly suggests that the hIgE mAb define multiple non-overlapping epitopes on Ara h 6.

## Discussion

Previous approaches to study human IgE antibodies have involved the use of chimeric antibodies with allergen-specific murine Fab regions fused to human IgE Fc regions (30, 31). Phage display techniques were used to generate Fab resulting from random combinations of human IgE heavy- and light-chain variable domains (32). These approaches produced IgE constructs that were useful for *in vitro* studies, but the synthetic antibodies (not naturally occurring) either used murine variable regions or did not necessarily have the natural pairing of human heavy and light chains that formed the IgE response in allergic people (33). The novelty of the hIgE mAb is that they are derived from symptomatic allergic patients and represent part of the human IgE repertoire of these patients (12). The clones reported are very rare, as indicated in Table 1 by the low IgE B-cell frequency (average of six IgE-positive cells per 10 million PBMC). Plasmablasts are not amenable to this approach because they do not grow in culture. Memory B cells have been reported to co-exist in blood with plasmablasts, with both types of cells encoding IgE (34). They appear to be sufficient to generate the large panel of IgE mAb reported here, which are representative antibodies from the human repertoire of allergic individuals. The ability to derive a broad range of full length naturally occurring hIgE mAb to a diverse array of allergens enables systematic dissection of allergen-specific IgE responses.

The hIgE mAb donors had symptoms of asthma, atopic dermatitis, or adverse reactions to foods. The antibody specificities from these patients broadly correlated with their allergic symptoms. A surprising outcome, due to the low frequency of IgE encoding B cells which results in low absolute numbers in small blood draws, was that hIgE mAb were obtained from young children, aged 3–10 years, who comprised 53% of the patients. Unsurprisingly, most of the hIgE mAb to food allergens were mostly seen in children. For young children, hIgE mAb were derived from only 1 to 10 ml of peripheral blood. The success of this approach was attributed to the expansion of IgE encoding B cells using irradiated NIH3T3 fibroblasts expressing CD40 ligand and secreting B-cell-activating factor and IL-21 (12). The increase of B cells in culture allows for greater rates of successful myeloma fusion and hybridoma generation. This is essential given the small quantity of blood that can be obtained and the extreme rarity of IgE encoding B cells.

The total IgE data provided convincing evidence that the hIgE mAb could be produced at concentrations that were orders of magnitude higher than those found in allergic patients. Clinically, CAP class 6 or ∼100 kU/L is considered a very high level of allergen-specific IgE (4, 23). The hIgE mAb levels were three to four logs higher and were comparable with those seen in patients with hyper-IgE syndromes or IgE myelomas. To compare the relative IgE activity of the different antibodies, the hIgE mAb were standardized at a total IgE level of ∼50,000 kU/L and then compared for specific IgE reactivity with purified allergen or with allergen extract. The hIgE mAb were obtained from highly productive hybridomas and showed remarkable levels of allergen-specific IgE, generally 2,000 to >50,000 kU/L: values that are much higher than those found in sera from allergic patients. In most cases, a good agreement between the allergen-specific and extract specific IgE was found. Some discrepancies between the levels of total IgE and allergen-specific IgE were noted. For ∼50% of hIgE mAb, the specific IgE levels ranged between 50% and 136% of the total IgE values. However, seven of the hIgE mAb had specific IgE levels that were <20% of the total IgE, including the hIgE mAb to alpha-gal, Der p 1, and Can f 6. Even in these cases, the actual levels of allergen-specific IgE significantly exceeded those found in sera from allergic patients. The reasons for differences between total and specific IgE levels are unclear. In some cases, the allergen-specific IgE values exceed those of the total IgE. Taken together, these results could reflect differences in IgE binding to the monoclonal anti-IgE antibodies used in the total IgE ImmunoCAP and the recombinant allergens or other proteins that are coupled to the solid phase for specific IgE measurements. To some extent, this is supported by the streptavidin CAP data for the hIgE mAb to peanut allergens, which showed lower binding to recombinant allergens but strong binding to natural allergens on the streptavidin CAP.

For the first time, the availability of hIgE mAb to food and inhaled allergens provides molecular probes to determine the structures of allergenic epitopes recognized by allergic patients. This transformative capability has recently been applied to determine the structure of the conformational epitope recognized by the 2F10 hIgE mAb on Der p 2 by x-ray crystallography. The 2F10 hIgE mAb, in combination with other hIgE mAb to Der p 2, passively sensitized human Fc*ε*RI*α*-transgenic mice and caused anaphylaxis: a rigorous demonstration of biological relevance (35). In the present study, *β*-hexosaminidase release from passively sensitized huRBL cells was used as an alternative approach to assess its biological activity. We recently published detailed comparisons of hIgE mAb potency in the huRBL system (27). In those studies, the hIgE mAb pairs to Der p 2, Fel d 1, and Ara h 2 produced high levels of mediator release and were directed against non-overlapping epitopes on each allergen. The current study showed high levels of *β*-hexosaminidase release using hIgE mAb pairs to Ara h 6. Similar huRBL data for the hIgE mAb to Ara h 2 was recently reported, and together these data support the concept that the hIgE mAb to food allergens provide screening tools for food allergy therapeutics (36).

The only epitope that was defined prior to this study was alpha-gal (20). Previously, the only available monoclonal antibodies to alpha-gal were a commercial murine IgM antibody (M86) and a mouse IgG1 antibody (27H8) (36, 37). The two hIgE mAb recognize alpha-gal on both bovine thyroglobulin and cetuximab. The hIgE mAb were from a 63-year-old patient who had severe reactions on several occasions after consumption of red meat and had a high total IgE and specific IgE to alpha-gal (P4, Table 1). Interestingly, immediately prior to these systemic allergic reactions to red meat, the patient was reported to have severe skin rash resulting from innumerable lower extremity bites of larval stage ticks (seed ticks). The hIgE mAb to alpha-gal will enable better diagnostic tests for alpha-gal to be developed. Investigations of the immunopathogenesis of red meat allergy, including the presence of alpha-gal in the tick saliva and the role of alpha-gal in coronary artery disease, will also be facilitated (38–42).

Limitations of this study are that hIgE mAb from a given individual may not represent the complete repertoire of IgE responses among the allergic population or that the fusion process followed by the antibody screening may select for certain epitope(s). Patients were randomly enrolled in these studies based on history, symptoms, and positive skin test to given allergens, rather than being targeted cohorts of a given age range or allergic sensitivity. In the future, pediatric and adult populations could be targeted for certain allergens, e.g., foods (peanut, milk, egg) or inhaled allergens (mite, cat, dog, pollens), to obtain a broader spectrum of the hIgE mAb. This would enable linear or conformational epitopes on food allergens to be investigated (12).

The hIgE mAb have several applications in the investigation of allergic diseases: first, as potential replacements for human sera and, second, as reference material(s) in allergy diagnostics. Access to the panels of the sera from allergic patients presents a significant barrier to innovation for diagnostic test developers (43). The hIgE mAb can serve as probes to identify specific allergens, as positive controls for current IgE tests and for the next generation of microarrays, nanotechnology, and point-of-care tests that are in the pipeline. The hIgE mAb have similar applications in cellular tests, such as the basophil activation test (BAT). The need for standardized reagents and procedures to validate the BAT and improve quality control has recently been emphasized (44).

Since the discovery of IgE, standardization of serologic measurements of IgE has relied on the World Health Organization’s human serum references for measuring the IgE levels for clinical diagnosis. The IgE tests produced by different manufacturers show variability and are not interchangeable (4). The hIgE mAb could ultimately be used to produce purified IgE reference standards in lieu of serum-based references as a calibrator of IgE measurements. This would be analogous to the National Institutes of Standards and Technology NISTmAb Reference Materials for validation of the methods used for the manufacture of therapeutic antibodies (45).

The unique value of the hIgE mAb to define allergenic epitopes recognized by allergic patients, to answer the question about what constitutes an allergenic epitope, will have applications in allergy therapeutics. A recent clinical trial demonstrated the efficacy of allergen-specific monoclonal antibodies to Fel d 1 to treat mild asthma (46). Mutations in allergen sequences can reduce anaphylaxis in mouse models, as we have shown for Der p 2 (35). The hIgE mAb will also have applications in mechanistic studies of IgE cross-linking and basophil and mast cell activation, all of which could lead to development of novel therapeutics. Improvements in allergy diagnostics and therapeutics using the hIgE mAb have the potential to transform the allergy field for the benefit of allergic patients.

## Data Availability

The original contributions presented in the study are included in the article/Supplementary Material, further inquiries can be directed to the corresponding author.
